# Superficial CD34-Positive Fibroblastic Tumor: A Case Series and a Review of Literature

**DOI:** 10.7759/cureus.48504

**Published:** 2023-11-08

**Authors:** Ghazi Zafar, Saira Nasir, Samina Zaman

**Affiliations:** 1 Histopathology, Chughtai Institute of Pathology, Lahore, PAK; 2 Pathology, Chughtai Institute of Pathology, Lahore, PAK; 3 Histopathology, Children Hospital and Institute of Child Health, Lahore, PAK

**Keywords:** superficial cd34-positive fibroblastic tumor, cd34 positivity, soft tissue tumours, immunohistochemistry, sarcoma

## Abstract

Superficial CD34-positive fibroblastic tumor (SCD34PFBT) is a recently recognized neoplasm of mesenchymal origin. Only a few cases have been reported in the literature so far. Microscopically, it consists of a dermal spindle cell neoplasm, with low mitotic activity, arranged in a fascicular pattern. The individual neoplastic cells show marked nuclear atypia, nuclear pseudo-inclusions, and dense eosinophilic cytoplasm. The tumor cells characteristically show positivity for CD34 immunohistochemical stain. This tumor behaves as a low-grade malignancy with potential to recur locally, with rare cases showing lymph node metastasis. Wide local excision and regular follow-up are the currently followed steps for management. This tumor serves as a diagnostic challenge due to its overt atypia, and it can be misdiagnosed as a sarcoma. Recognition of this entity among pathologists is important due to this reason. We hereby report four cases of this newly recognized entity.

## Introduction

Superficial CD34-positive fibroblastic tumor (SCD34PFBT) is a recently recognized neoplasm of mesenchymal origin, first described in 2014 [1]. As the name implies, this tumor is superficially located and shows positivity for CD34 on immunohistochemistry. Microscopically, the tumor is composed of cellular fascicles of spindled to epithelioid neoplastic cells, with marked cellular pleomorphism, bizarre morphology, nuclear pseudo-inclusions, and prominent nucleoli [2]. The tumor has been incorporated into the new fifth edition of the World Health Organization (WHO) classification of soft tissue tumors [3]. It is important to recognize this entity, as it may be misdiagnosed as a high-grade sarcoma owing to its marked nuclear pleomorphism. SCD34PFBT is a locally aggressive neoplasm, with resection being the preferred treatment of choice [4,5]. In this case series, we will be discussing four cases of SCD34PFBT along with a review of recent and emerging literature.

## Case presentation

Case 1

A 38-year-old female presented with a mass on the left side of the left chest. On clinical examination, the mass was found to be superficial in nature, located in the subcutaneous tissue. Clinical differential diagnosis included accessory breast tissue, benign adnexal neoplasm, and fibroadenoma. Excision of the mass was performed.

We received an excision specimen measuring 60 x 49 x 42 mm with overlying tan brown unremarkable ellipse of the skin measuring 57 x 45mm, without any ulceration. Cut section showed a relatively circumscribed firm yellowish mass measuring 40 x 37 x 30 mm, which was 1 mm away from the closest resection margin.

**Figure 1:**
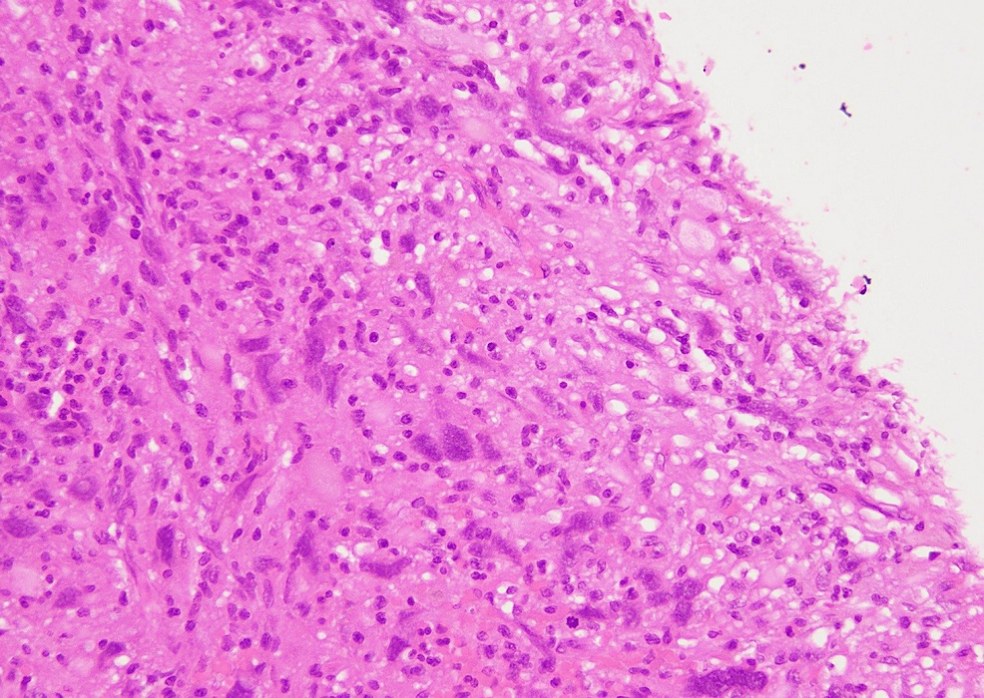
Tumor cells with marked nuclear pleomorphism (H&E stain, 40X) H&E, hematoxylin and eosin

The microscopic examination of the mass revealed a circumscribed spindle cell neoplasm, arranged in fascicles, located in the deep dermis and subcutaneous tissue. The neoplastic cells had a marked degree of nuclear pleomorphism with intranuclear pseudo-inclusions (Figure 1).

**Figure 2:**
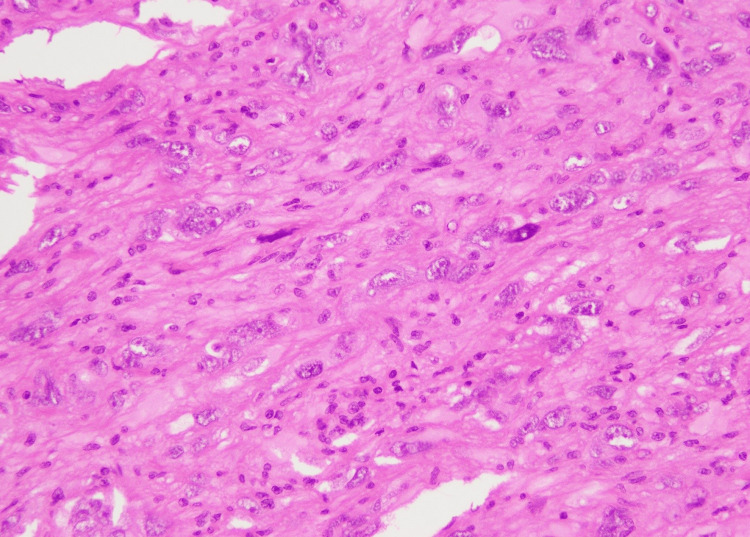
The tumor background showed inflammatory cells comprising predominantly lymphocytes (H& stain, 40X) H&E, hematoxylin and eosin

At the advancing edge, lipidized cells with vacuolated foamy cytoplasm were seen. The mitotic activity was very low (one to two mitotic figures per 20 high power fields [HPF]). The background was rich in inflammation comprising lymphocytes and a few eosinophils (Figure 2).

**Figure 3:**
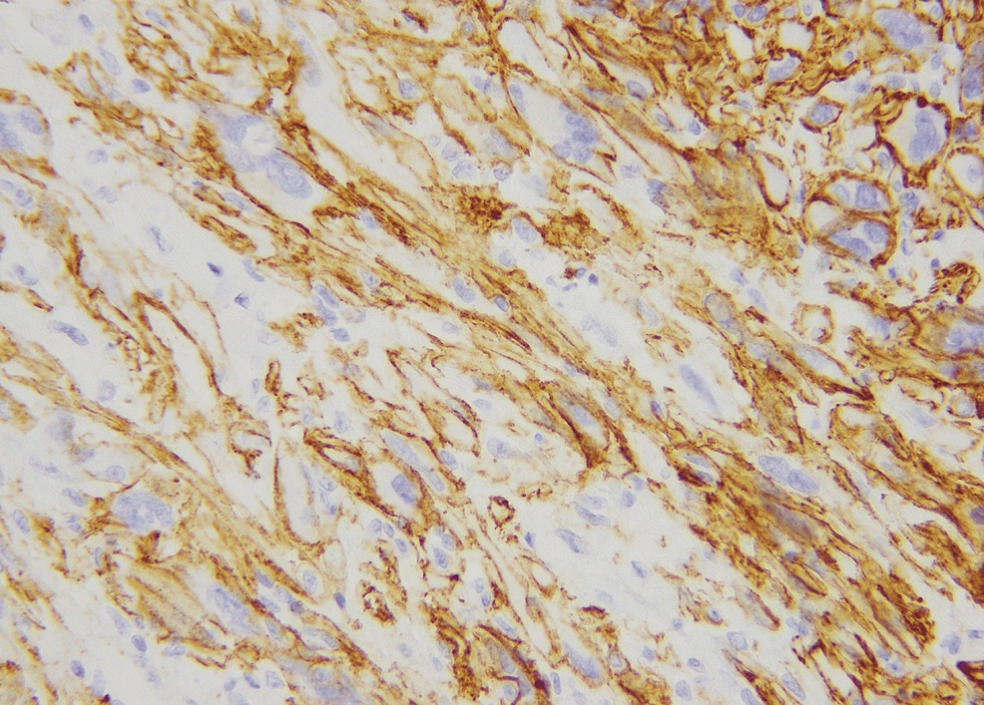
Membranous positivity for CD34 immunohistochemical stain (40X)

No atypical mitotic figure was seen, and there was no necrosis. The excision was complete. The histologic differential diagnosis included SCD34PFT, atypical fibrous histiocytoma, poorly differentiated carcinoma, leiomyosarcoma, and undifferentiated pleomorphic sarcoma. Extensive immunohistochemical workup was done, and the neoplastic cells were diffusely and strongly positive for CD34 and D2-40 immunohistochemical stains (Figure 3).

Smooth muscle actin (SMA) was also focally positive. While pan-cytokeratin (AE1/AE3), S-100, leukocyte common antigen (LCA), anaplastic lymphoma kinase (ALK), desmin, human melanoma black 45 (HMB45), CD68, and mouse double minute 2 (MDM-2) were negative. The final diagnosis of SCD34PFBT was made. The patient was followed with regular monthly radiological assessment for the first year and then radiology after six months. After 18 months of follow-up, no metastasis or local recurrence was found.

Case 2

A 41-year-old male presented with a slowly increasing subcutaneous soft tissue mass on the right forearm since many years. Clinical suspicion of lipoma was raised and it was excised.

**Figure 4:**
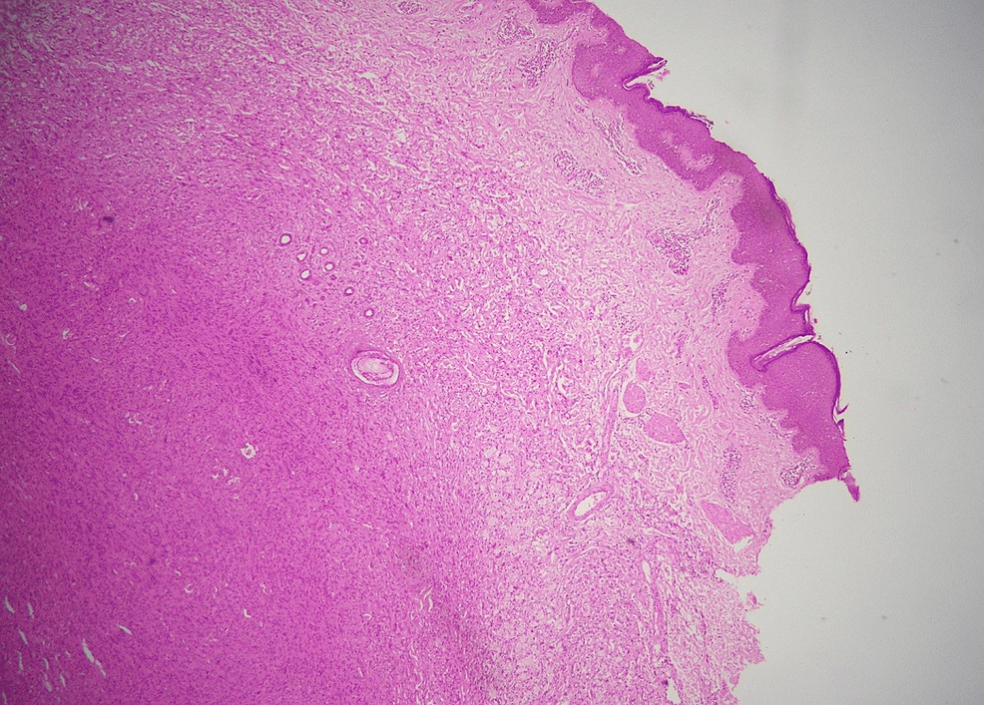
Dermal location and circumscribed nature of the tumor (H&E stain, 4X) H&E, hematoxylin and eosin

We received an excision specimen measuring 28 x 21 x 10 mm. Overlying skin ellipse measured 28 x 21 mm. Cut section revealed a yellowish tumor measuring 27 x 21 mm, which grossly involved inked margin. The microscopic examination revealed skin with underlying subcutaneous tissue, showing a circumscribed spindle cell neoplasm (Figure 4).

**Figure 5:**
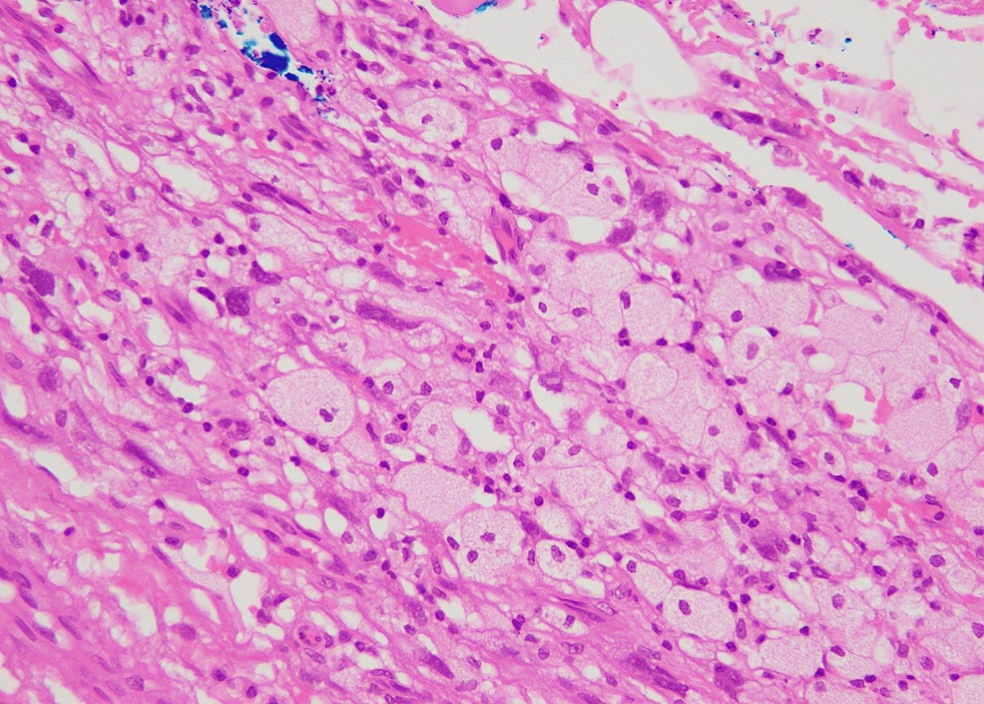
Lipidized cells at the advancing edge (H&E stain, 40X) H&E, hematoxylin and eosin

At the advancing edge of the tumor, lipidized cells with foamy cytoplasm were noted (Figure 5).

**Figure 6:**
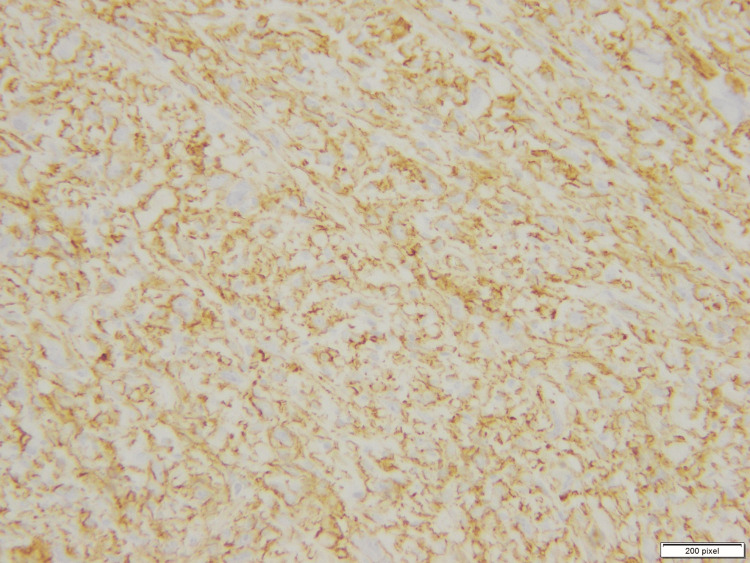
Membranous positivity of CD34 immunohistochemical stain (20X)

**Figure 7:**
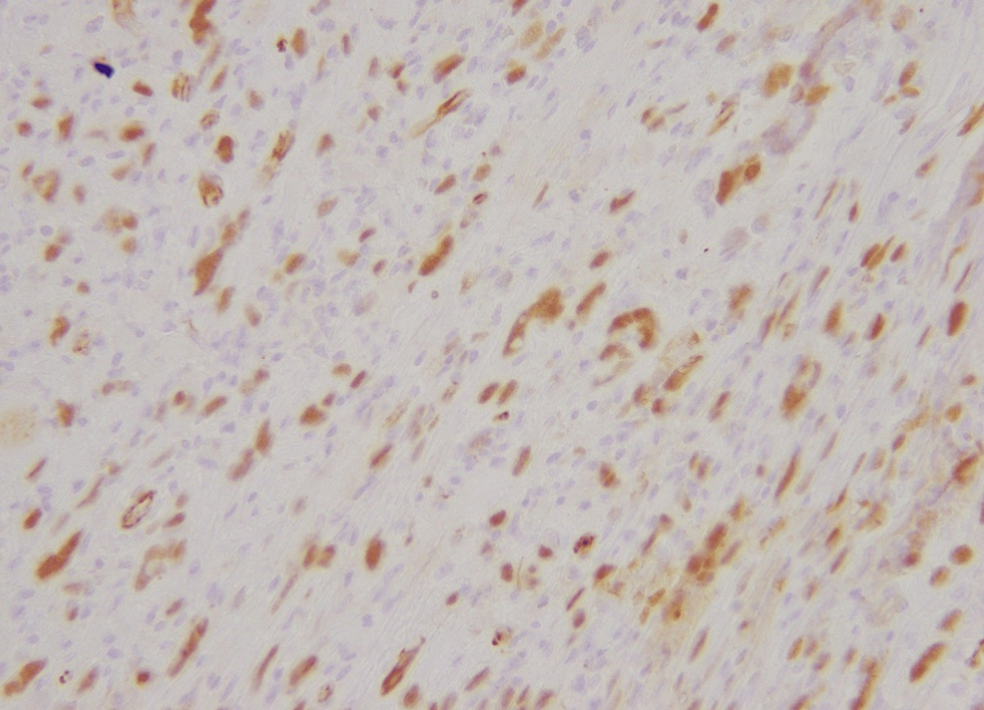
Nuclear positivity of WT-1 immunohistochemical stain in tumor cells (40X)

The spindle cells show marked nuclear pleomorphism, with prominent nucleoli and intranuclear inclusions. The mitotic activity was one to two mitotic figures per 10 HPF. No atypical mitosis or areas of necrosis were seen. The tumor involved inked resection margin. The histologic differential diagnosis included SCD34PFT, leiomyosarcoma, and undifferentiated pleomorphic sarcoma. The immunohistochemical workup showed positivity for CD34 (Figure 6) and WT-1 (Figure 7).

**Figure 8:**
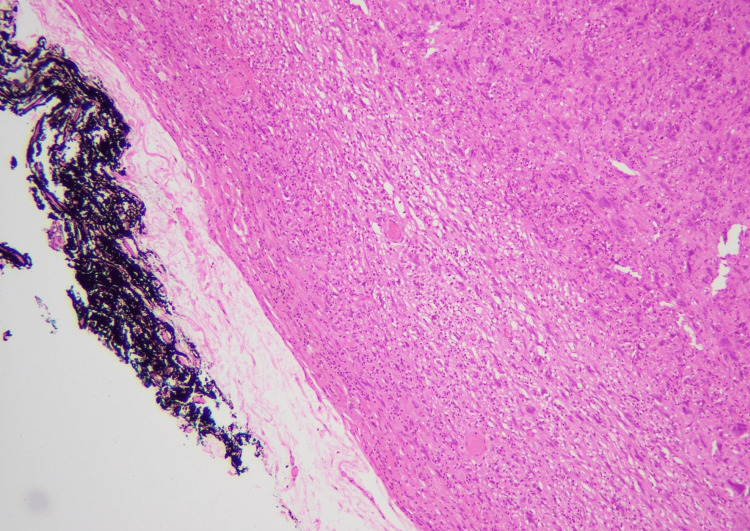
Re-excision specimen with clear (black) inked margin (20X)

SMA, pan-cytokeratin (AE1/AE3), S-100, p53, and desmin were negative. The final diagnosis of SCD34PFBT was made. Margin re-excision was performed, which came out to be negative (Figure 8).

After three months of the re-excision, MRI was performed, which showed no recurrence. The six-month follow-up is awaited.

Case 3

A 20-year-old male presented with a painless swelling left upper thigh for five years. The mass progressively increased in size. On radiology, the mass was located within soft tissue only, without bony extension. Clinical differentials included lipoma, liposarcoma, and sarcoma. Wide local excision of the mass was performed.

We received a wide local excision specimen measuring 90 x 70 x 30 mm. Overlying skin ellipse measured 90 x 50 mm. Cut section revealed a tumor measuring 70 x 50 x 30 mm. Grossly all margins were free, with the closest margin being free by 1 mm.

**Figure 9:**
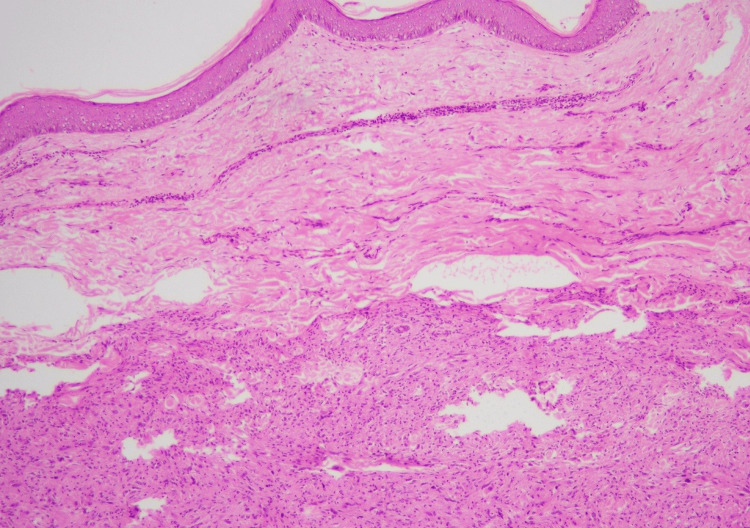
Circumscribed dermal neoplasm in a fascicular pattern (H&E stain, 10X) H&E, hematoxylin and eosin

The microscopic examination of the sections from the tumor revealed skin with underlying tissue, showing a circumscribed deep dermal spindle cell neoplasm arranged in a fascicular pattern (Figure 9).

**Figure 10:**
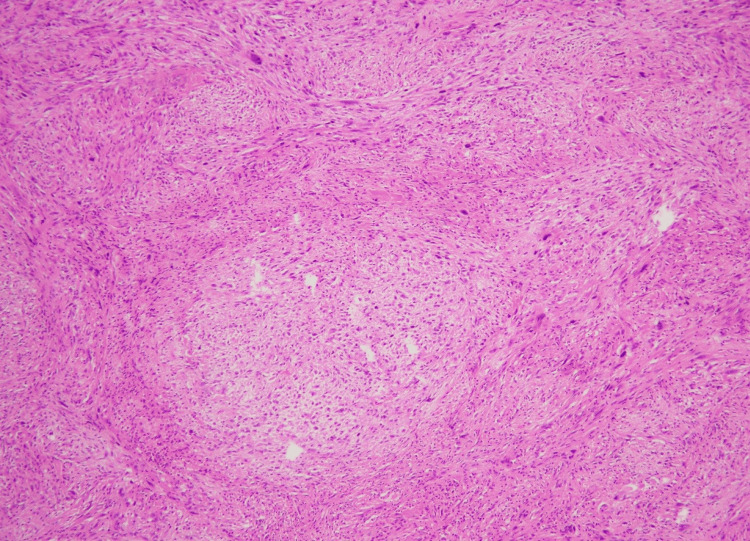
Marked nuclear pleomorphism (H&E stain, 10X) This image shows obvious pleomorphism even at low magnification H&E, hematoxylin and eosin

The spindle cells show elongated nuclei and eosinophilic cytoplasm. Some cells show a marked degree of nuclear pleomorphism, prominent nucleoli, and intranuclear inclusions (Figure 10).

**Figure 11:**
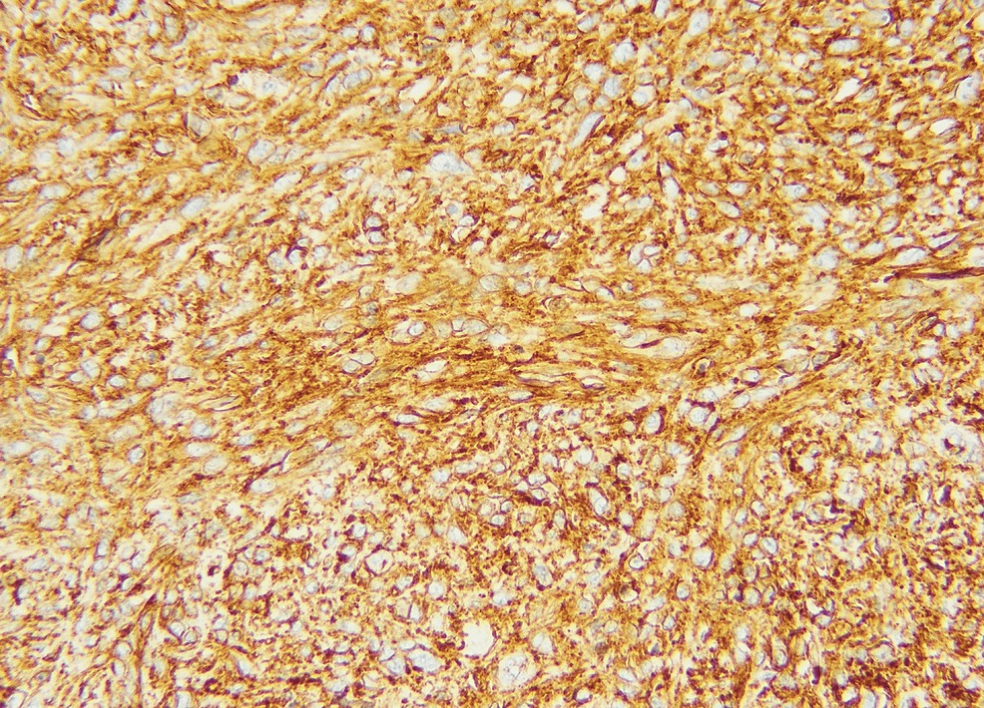
Strong membranous CD34 immunohistochemical expression (20X)

**Figure 12:**
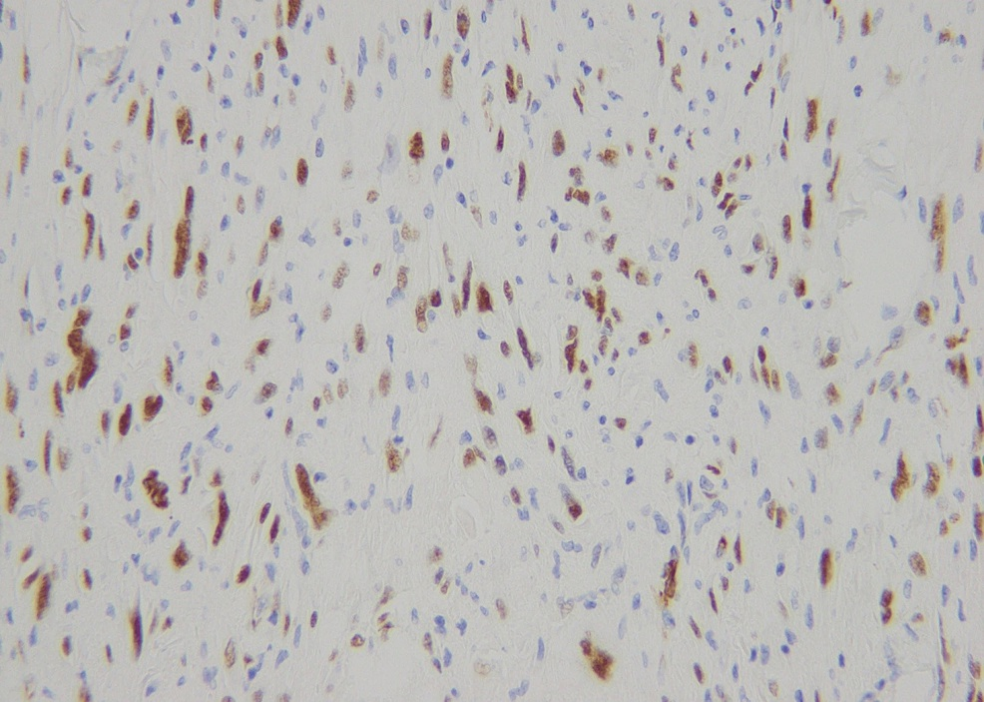
Weak nuclear positivity for WT-1 immunohistochemical stain (40X)

Focally increased mitoses were noted up to two to three mitotic figures per 10 HPF. However, no atypical mitotic figures or areas of necrosis were seen. The tumor was predominantly circumscribed, but some infiltration was noted. The margins were free. The immunohistochemical workup showed positivity for CD34 (Figure 11) and weak nuclear expression of WT-1 (Figure 12).

SMA, S-100, desmin, MDM-2, ALK, and CD68 were all negative. The final diagnosis of SCD34PFBT was made. So far, no recurrence has been noted at the six-month follow-up of the patient.

Case 4

A 40-year-old male presented with a freely mobile swelling in the anterior compartment of the left leg for the last 10 years. Initial fine needle aspiration cytology performed at an outside laboratory showed inflammatory and degenerated cells only. MRI was performed, which revealed a 79 x 75 x 55 mm oval shaped well-circumscribed mass anterior to the sartorius and rectus femoris muscle. Clinical differential diagnosis of a soft tissue sarcoma and lipoma was made.

We received an excision specimen measuring 80 x 80 x 60 mm. Cut section revealed a neoplasm with diffuse whitish cut surface. Grossly all margins were free, with the closest margin being free by 1 mm.

**Figure 13:**
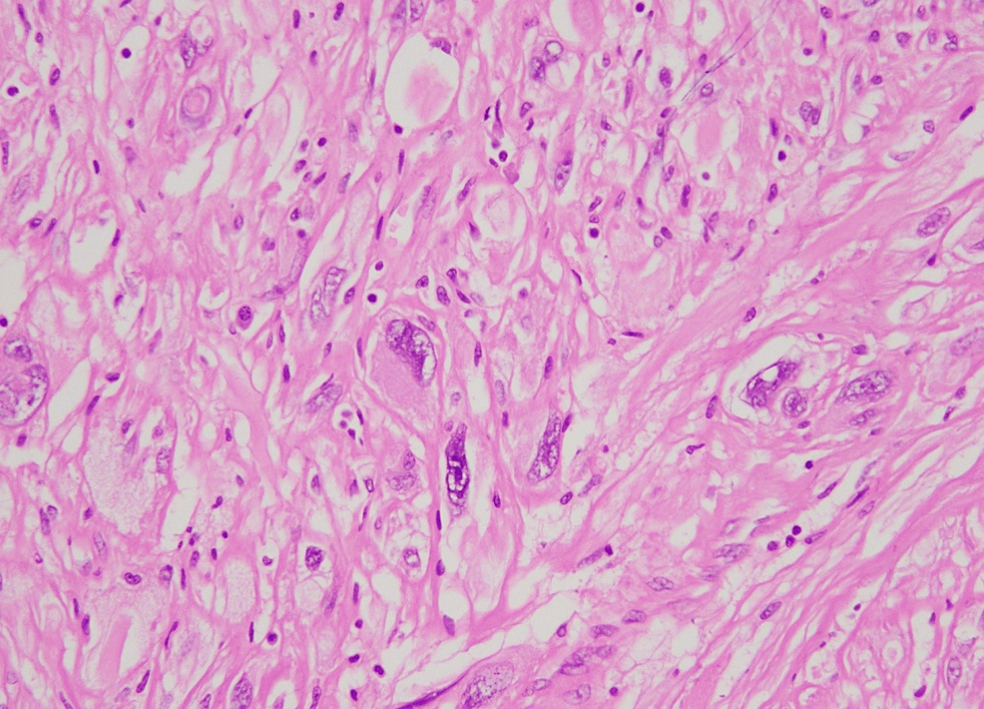
Marked nuclear atypia with nuclear pseudo-inclusions in tumor cells (H&E stain, 40X) H&E, hematoxylin and eosin

**Figure 14:**
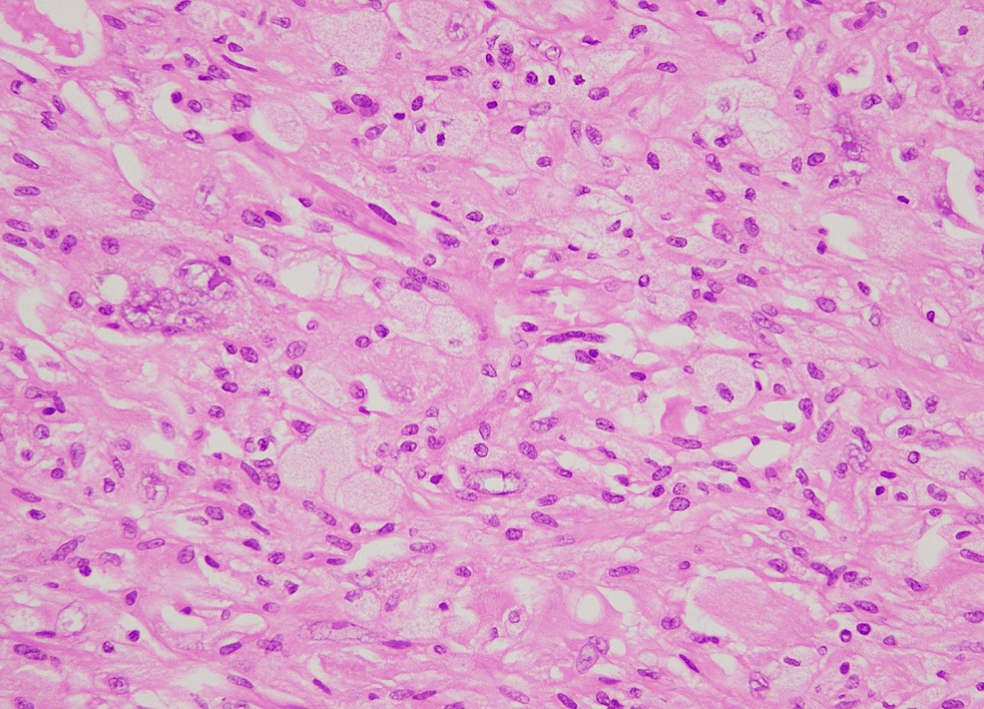
Lipidized cells, predominantly at the advancing edge (H&E stain, 40X) H&E, hematoxylin and eosin

The microscopic examination of the tumor revealed a dermal circumscribed cellular tumor arranged in fascicles of spindled to epithelioid cells. The cells show markedly bizarre pleomorphic nuclei with nuclear pseudo-inclusions and glassy eosinophilic cytoplasm (Figure 13). Many lipidized cells were also noted (Figure 14).

**Figure 15:**
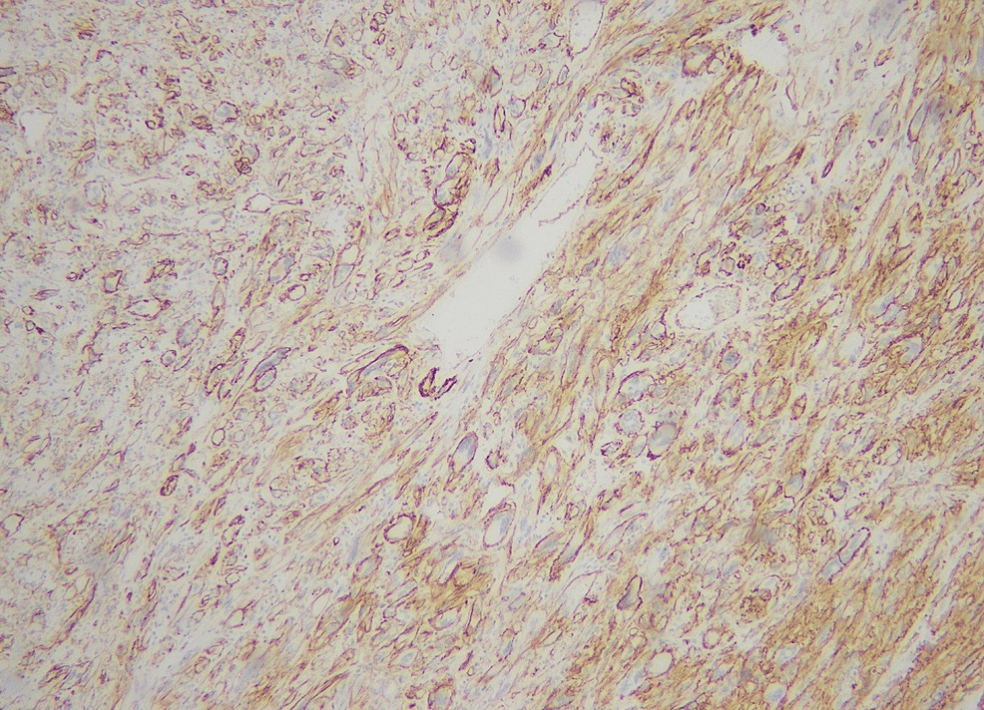
Positive CD34 stain (20X)

**Figure 16:**
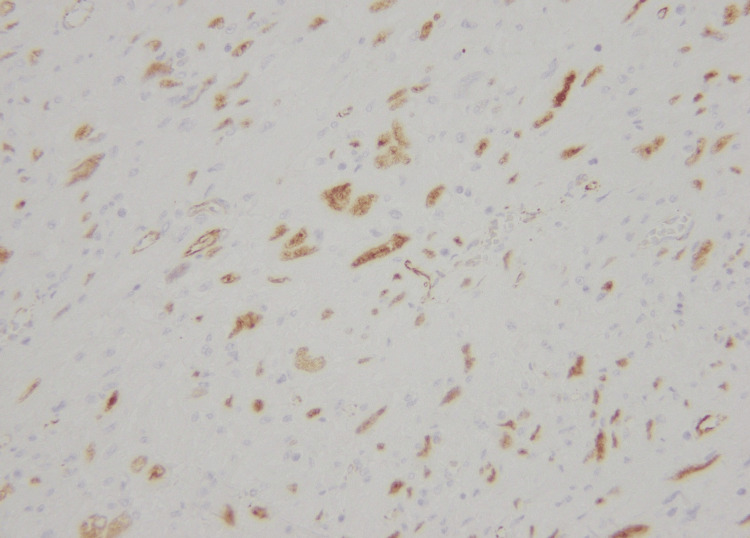
Nuclear expression of WT-1 immunohistochemical stain (40X)

**Figure 17:**
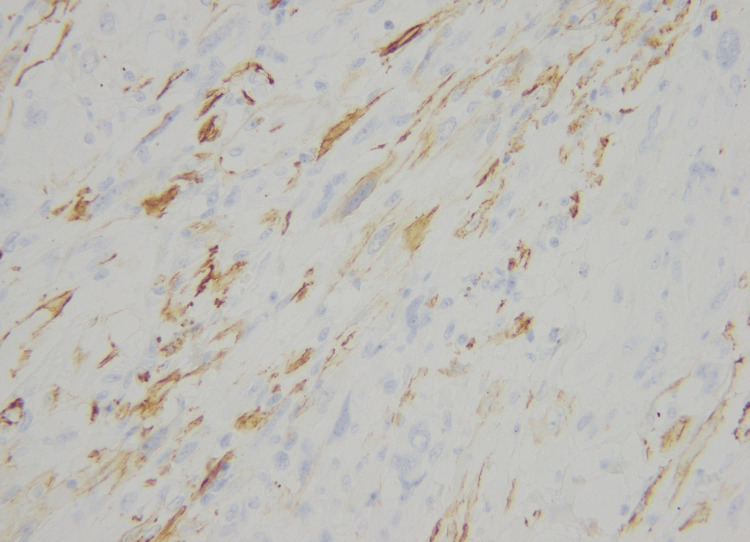
Weak expression of smooth muscle actin in the tumor cells (40X)

There was a moderate degree of acute on chronic inflammatory cell infiltrate in the background, which was lymphocytic predominant. Hemosiderin-laden macrophages were also seen. The tumor only showed one mitosis per 10 HPF. The tumor cells were diffusely positive for CD34 (Figure 15) and WT-1 (Figure 16). The tumor cells showed weak membranous staining for SMA and caldesmon as well (Figure 17).

However, desmin, cytokeratin, SOX-10, and MDM-2 were negative. The final diagnosis of SCD34PFBT was made. The patient attended two follow-up visits with radiological assessment at an interval of six months each. No tumor recurrence or metastasis has been observed.

## Discussion

Soft tissue neoplasms of fibroblastic and myofibroblastic origin are categorized as benign, intermediate, and malignant depending on their clinical behavior, local recurrence, and metastatic potential. SCDPFBT is classified as an intermediate-grade (low-grade), rarely metastasizing tumor [3,6]. To date, less than 100 cases have been reported, owing to its rarity along with lack of familiarity and recognition among pathologists [7].

SCD34PFBT commonly occurs in the fourth and fifth decades of life, with predilection to the lower limb, and frequently presents as a slow-growing painless subcutaneous mass. Upon the review of literature, we found a series published by Anderson et al. in 2022 discussing the clinicopathological, immunohistochemical, and molecular findings of 59 cases [7]. In this series, it was noted that the tumor most commonly arises in the lower limb (73%), with almost equal gender distribution (male:female ratio = 1.27:1), median age of 42 years, and median tumor size of 3.0 cm (range: 1.0 cm to 9.0 cm). Another series of 11 cases published by Lao et al. also reported the lower limb to be the most common site (7/11, 64%) [8]. Male predominance was also noted (8/11, 73%). In their series, the median age was 36 years; however, a male predominance was noted. In our cases, the mean age was 35 years, with two cases in the lower limb (50%). Male predominance was noted in our study as well. Our findings are consistent with both of these series.

Microscopically, SCD34PFBT is located in the deep dermis and subcutaneous tissue and is generally well-circumscribed. Limited invasion into the adjacent subcutaneous fat can be seen [9]. The tumor cells are spindled to the epithelioid, usually arranged in fascicles, with dense eosinophilic cytoplasm. The nuclei are often bizarre appearing with frequent intranuclear inclusions. Generally, mitotic activity is low and no necrosis is seen. Mixed inflammation comprising lymphocytes, plasma cells, and occasional neutrophils is a common finding. Lipidized cells along with areas of hemosiderin deposition are also commonly appreciated in the tumor.

Immunohistochemically, the tumor is seen to express CD34 stain consistently. Other stains that are known to express variably comprise WT-1, CK AE1/AE3, and Cam 5.2. Our cases also express CD34 consistently, while WT-1 expression was also appreciated, at least focally. One of our cases also expressed a D2-40 stain. As per our literature review, this finding has not been noticed. Weak, patchy expression of SMA and caldesmon was also appreciated in one of our cases. Recently, CADM3 along with cyclin D1 and SynCam3 immunohistochemical stains have emerged to be specially expressed in SCD34PFBT, when compared with its usual differential diagnosis [10,11]. According to various studies, a subset of SCD34PFBT harbors PRDM10 rearrangements [10].

The differential diagnosis in case of SCD34PFBT are wide and include both benign and highly malignant tumors. Usually, the differentials considered include fibrohistiocytic neoplasms including dermatofibrosarcoma protuberans (DFSP), owing to their CD34 positivity. Other differentials that should be considered include undifferentiated pleomorphic sarcoma, leiomyosarcoma, pleomorphic dermal sarcoma, epithelioid sarcoma, solitary fibrous tumor, de-differentiated liposarcoma, melanoma, and anaplastic large cell lymphoma. Microscopic features including bizarre cells with nuclear pseudo-inclusions, lack of increased mitoses, and lack of necrosis along with diffuse strong expression of CD34 often lead to correct diagnosis, provided that SCD34PFBT is considered in the differentials [1,2,7-9].

Usual treatment option for SCD34PFBT is total surgical excision. In the study of Anderson et al., only two cases demonstrated local recurrence (6%), while one case had distant lymph node metastasis [7]. SCD34PFBT is a recently recognized entity; thus, updates regarding its clinical behavior are still evolving. Long-term data regarding clinical behavior are also lacking. Therefore, it is usually recommended to have a long-term follow-up of the patients.

## Conclusions

We reported four cases of a recently recognized entity, SCD34PFBT. All of these four cases were reported within the last two years. This proves that this entity is under-reported, rather than extremely rare. Characteristic microscopic features on bizarre tumor cells with nuclear pseudo-inclusions, low mitotic activity, lack of necrosis, presence of lipidized cells, and diffuse expression of CD34 immunohistochemical stain must raise the concern of this entity. This tumor has been misdiagnosed as a sarcoma in the past, leading to overtreatment of the patient. However, it has guarded behavior with only rare instances of lymph node metastasis. Long-term behavior of the entity is still evolving.
